# The composition of braconid wasp communities in three forest fragments in a tropical lowland forest of Panama

**DOI:** 10.1186/s12862-022-02051-4

**Published:** 2022-08-13

**Authors:** Louise A. Rodríguez, Enrique Medianero

**Affiliations:** 1 Programa de Maestría en Ciencias Biológicas, Universidad de Panamá, Campus Octavio Méndez Pereira, Avenida Transístmica, Panama City, Panama; 2 Departamento de Ciencias Ambientales and Programa de Maestría en Entomología, Universidad de Panamá, Campus Octavio Méndez Pereira, Avenida Transístmica , Panama City, Panama; 3 Miembro del Sistema Nacional de Investigación (SNI-SENACYT), Panama City, Panama

**Keywords:** Braconidae, Forest fragments, Parasitoid, Similarity Index

## Abstract

**Background:**

In the last 171 years, the forests along the eastern bank of the Panama Canal have been pressured by anthropic activities. Studies of the influence of habitat fragmentation on braconid wasp communities in Central America is scarce, showing the existing information gap on these communities required to implement strategic plans for ecosystem sustainability and conservation. This study investigated how fragmentation affects braconid wasp communities in three areas in Panama City: Metropolitan Natural Park, Albrook and Corozal. Two permanent Malaise Traps were installed in the center of each fragment and were reviewed weekly from May 2019 to March 2020. Alpha and beta diversity indices and the similarity index were used to demonstrate the composition of braconid wasp communities in three forest fragments.

**Results:**

A similarity of 94% was estimated for the subfamily composition and 74% was estimated for the morphospecies composition of wasp community in the fragments studied. Wasp subfamily and morphospecies assemblages were more similar between fragments of Albrook and Metropolitan Natural Park. Richness and abundance of braconid wasps observed were statistically different between the fragments studied.

**Conclusion:**

Richness, abundance, and composition of braconid wasps differ among habitat fragments with high similarity between subfamilies and morphospecies. Therefore, the fragments studied can be used as stepping stones to maintain remaining populations of braconid wasp communities. Monitoring is recommended to assess the effect of fragmentation on the remaining forests.

## Background

Habitat fragmentation represents one of the most serious threats to global biodiversity [1–6]. Global assessments have shown that habitat fragmentation is provoking a decrease in population size and an increased risk of disappearance of many flora and fauna [7, 8]. More than 50% of tropical forests have been degraded globally and with the use of satellite images, it is revealed that almost 43% of the terrestrial surface has been converted from its natural state for anthropogenic purposes [9, 10]. From 2000 to 2010, there was a net loss of forest cover of 7 million hectares (ha) per year in the tropical countries of the world [11]. In Panama, the forest cover has decreased from 5,245,000 ha in 1947 to 2,481,658 ha in 2019, which represents 47% forest cover loss [12]. If the fragmentation process continues at an exponential rate, the world’s tropical forests could disappear completely [13].

Habitat fragmentation often occurs due to some disturbance mechanism (agriculture, deforestation, urbanization, fires, etc.) as a result of topographic differences [14]. As the world’s human population continually increases, urban areas are growing rapidly and threaten the habitat of native species of flora and fauna. Studies investigating the influence of habitat fragmentation on biodiversity and the risk of species extinction are of the utmost importance and have been a main focus of biodiversity conservation research [15–20]. For instance, the abundance of dung beetle decreased with increased urban land cover [21]. Both common and rare species of social wasps are threatened by forest fragmentation in Central Amazon [22].

In tropical forests, evidence indicates that one of the taxa that respond faster to environmental changes are insects [23, 24]. Insects play important roles in almost all trophic levels; therefore, it is important to understand the response of these organisms to fragmentation. Within this taxon, parasitoids as a group may be used to assess the effects of habitat fragmentation because they play an ecological role in regulating populations of other insects due to prey denso-dependence [13]. González and Ruíz [25] proposed the use of braconid wasps (parasitoids) as indicators of biological diversity in deciduous forests and in evaluating and monitoring the effects of anthropogenic activities on ecosystems. Braconid wasps are regulatory agents of various groups of herbivorous insects that indicate the presence or absence of other species through the food chain [26]. Most braconid wasps are endo and exo parasitoids which feed on the larval stages of Coleoptera, Diptera and Lepidoptera [27]. This makes braconid wasps good biological indicators of habitat disturbances [25].

Smith and Mayfield [28] studied the taxonomic and functional diversity of bees visiting flowers of three tree species in small and large tropical forest fragments in tropical Australian landscapes. Species and functional diversity were found to differ significantly between small and large fragments. There was less taxonomic diversity of bees visiting flowers in small fragments. Additionally, native eusocial stingless bees were not common on small remains despite the presence of floral resources similar to those sampled on large remains [28].

In a similar study, Ruiz-Guerra et al. [29] studied the abundance, species richness, similarity, and prevalence of braconid parasitoid wasps for four types of land use (secondary forest, plantations, live fences, and pastures) and preserved tropical humid forest remnants in southern Mexico. Species richness and abundance were found to be higher in preserved and secondary forests than in other land use types.

To establish more direct links with ecosystem processes, there is a need to investigate patterns of functional trait and taxonomic diversity with biological indicators. Fragmentation experiments are useful tools used to provide clear evidence of the strong and typically degrading impacts of biodiversity loss [30]. Studies on the influence of habitat fragmentation on braconid wasp communities in lowland forests of Central America are scarce, portraying the existing information gap on these communities required to implement strategic plans for ecosystem sustainability and conservation. It is expected that landscapes with an intermediate degree of fragmentation will cause separation of braconid wasp communities which is reflected by a low similarity between communities. This research study contributes to the construction of baseline data by evaluating the vulnerability of braconid wasp communities leading to strategic plans for the sustainability and conservation of ecosystems. We sought to determine how habitat fragmentation may affect braconid wasp communities in fragmented lowland forest locations in Panama using similarity and fragmentation indices.

## Results

**Fig. 1:**
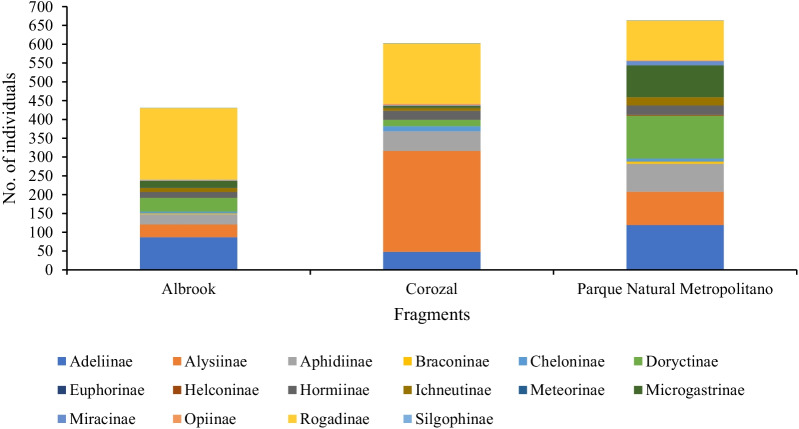
Variation in individual abundance of braconid wasp in the three habitat fragments studied in lowland tropical forests of Panama. Each panel shows data for a habitat fragment and is grouped by subfamily. Stacked bar plots show the dominant subfamilies within each habitat fragment

A total of 1697 individual wasps belonging to 77 morphospecies and 16 subfamilies were recorded. Of the 1697 individuals, approximately 39% were collected in the PNM fragment, 36% in the fragment of COR and 25% in the fragment of ALB (Table 1). Among the three fragments, Rogadinae was found to be the most abundant subfamily with 456 individual wasps, followed by Alysiinae with 391 individual wasps, Adeliinae with 254 individual wasps, Doryctinae with 168 individual wasps, Aphidiinae with 154 individual wasps and Microgastrinae with 108 individual wasps. In the PNM fragment, 664 individual wasps and 14 subfamilies were observed (Table 1); with the most abundant subfamily being Adeliinae with 119 individual wasps, followed by Doryctinae with 114 individual wasps, Rogadinae with 106 individual wasps, Alysiinae with 89 individual wasps and Microgastrinae with 85 individual wasps (Fig. 1). In the COR fragment, 603 individual wasps and 13 subfamilies were observed (Table 1); with the most abundant subfamily being Alysiinae with 268 individual wasps, followed by Rogadinae with 160 individual wasps, Aphidiinae with 53 individual wasps and Adeliinae with 48 individual wasps (Fig. 1). In the ALB fragment, 430 individual wasps and 16 subfamilies were observed (Table 1); with the most abundant subfamily being Rogadinae with 190 individual wasps, followed by Adeliinae with 87 individual wasps, Alysiinae with 34 individual wasps and Aphidiinae with 27 individual wasps (Fig. 1).

**Table 1:** Numbers of subfamilies, morphospecies and individuals of braconid wasps in the three fragments studied during the years 2019–2020

Site	No. of morphospecies	No. of subfamily	No. of individuals	% of individuals
PNM	56	14	664	39.1
COR	44	13	603	35.5
ALB	52	16	430	25.3

The results indicate a greater number of morphospecies in Metropolitan Natural Park (PNM), of subfamilies in Albrook (ALB) and of individuals in Corozal (COR)

Among the three fragments, the most abundant morphospecies belonged to the subfamilies Rogadinae (M 112 y M1), Adeliinae (M 117), Microgastrinae (M 118), Aphidiinae (M 130) and Alysiinae (M 152), respectively (Table 2). The aforementioned morphospecies account for approximately 65% of the total individual wasp separated as morphospecies. In the PNM fragment, the most abundant morphospecies belonged to the subfamilies Rogadinae (M 117) and Microgastrinae (M 118) with 80 individual wasps for each, followed by Alysiinae (M 152) with 75 individual wasps, Doryctinae (M 128) with 73 individual wasps and Aphidiinae (M 130) with 73 individual wasps (Table 2). In the COR fragment, the most abundant morphospecies belonged to the subfamilies Rogadinae (M 112, M 1 and M 29) with 387 individual wasps and Aphidiinae (M 113) with 32 individual wasps (Table 2). In the ALB fragment, the most abundant morphospecies belonged to the subfamilies Rogadinae (M 1, M117, M5 and M2) with 215 individual wasps and Adelinae (M 116) with 21 individual wasps (Table 2).

**Table 2:** Abundance and relative abundance of morphospecies in the three fragments studied during the years 2019–2020

Morphospecies no. (subfamily)	Total	ALB	COR	PNM
112 (Rogadinae)	286	20	265	1
1(Rogadinae)	230	91	93	46
117 (Adeliinae)	162	53	29	80
118 (Microgastrinae)	97	16	1	80
130 (Aphidiinae)	91	16	21	54
128 (Doryctinae)	89	12	4	73
152 (Alysiinae)	89	11	3	75
113(Aphidiinae)	63	11	32	20
5 (Rogadinae)	60	40	20	0
116 (Adeliinae)	59	21	12	26
2 (Rogadinae)	40	31	5	4
119 (Icheutinae)	40	11	7	22
4 (Rogadinae)	39	10	24	5
3 (Rogadinae)	34	8	3	23
68 (Braconinae)	22	0	14	8
121 (Cheloninae)	18	3	12	3
139 (Adeliinae)	17	7	2	8
120 (Adeliinae)	16	6	5	5
137 (Alysiinae)	16	3	0	13
144 (Doryctinae)	15	3	0	12
153 (Hormiinae)	15	3	2	10
19 (Miracinae)	14	1	2	11
138 (Doryctinae)	14	3	4	7
140 (Rogadinae)	12	1	4	7
51 (Hormiinae)	11	9	0	2
52 (Hormiinae)	11	1	8	2
154 (Rogadinae)	10	1	3	6
50 (Rogadinae)	9	1	3	5
151 (Doryctinae)	9	2	1	6
20 (Doryctinae)	8	3	2	3
66 (Braconinae)	8	2	0	6
31 (Cheloninae)	6	2	1	3

The table shows 95% of the data for the total individual wasps found

The Margalef index value indicated a higher species richness in the PNM, followed by ALB and COR, respectively (Table 3). The range of D´ values were close to 1 in the three fragments studied, which represents a lower degree of dominance/predominance of part of one or two species, which is also interpreted as a high diversity (Table 3). The values found with this index showed that the fragments with the highest diversity are ALB and PNM and that of COR showed a lower diversity. The H´ index indicated that the fragments with the greatest diversity were ALB and PNM and the lowest value was found in the COR fragment. According to the range of J´values all the fragments were proportional to the diversity and those that presented a more equitable distribution/pairing were ALB and PNM (Table 3). The fragment that presented less equality was COR.

**Table 3:** Indices of α diversity of braconid wasps in three habitat fragments in Panama

	ALB	COR	PNM
Simpson_1-D	0.91	0.77	0.90
Shannon_H	2.95	2.24	2.87
Margalef	7.92	6.25	8.00
Equitability_J	0.76	0.60	0.72

According to the Whittaker index, the highest turnover value in the composition of morphospecies recorded was between the COR and PNM fragments, followed by COR and ALB, and finally the ALB and PNM fragments (Table 4). The highest turnover value in the composition of subfamilies recorded was between the COR and PNM fragments, followed by COR and ALB, and finally the ALB and PNM fragments (Table 5).

**Table 4:** Whittaker’s β diversity index of morphospecies of braconid wasps in three habitat fragments in Panama

	ALB	COR	PNM
ALB	0	0.356	0.275
COR		0	0.383
PNM			0

**Table 5:** Whittaker’s β diversity index of subfamilies of braconid wasps in three habitat fragments in Panama

	ALB	COR	PNM
ALB	0	0.103	0.067
COR		0	0.111
PNM			0

**Fig. 2:**
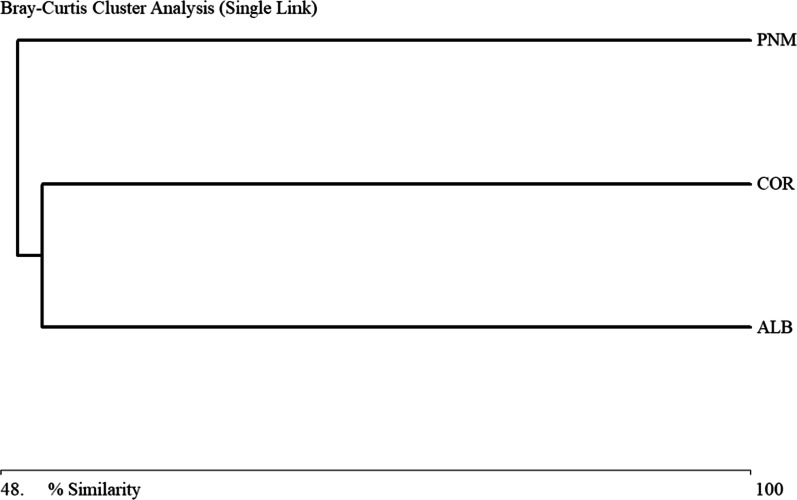
Similarity dendrogram (Bray–Curtis) of the Braconidae morphospecies found in the three fragments studied

**Fig. 3:**
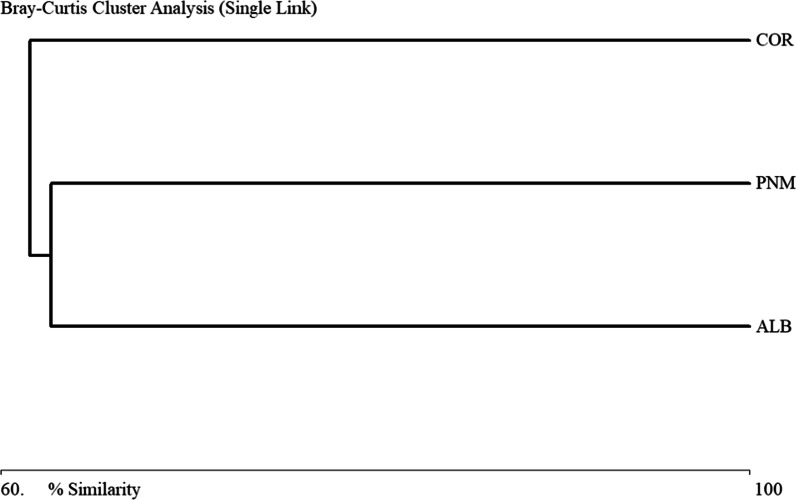
Similarity dendrogram (Bray–Curtis) of the Braconidae subfamilies found in the three fragments studied

The Bray–Curtis similarity analysis showed a morphospecies similarity and variation of approximately 0.5706 among the three fragments studied (Fig. 2). In the dendrogram it can be seen that there are two groupings where the morphospecies composition is more similar between the COR and ALB fragments with 0.51 (Fig. 2). The Bray–Curtis similarity analysis showed a similarity and variation of subfamilies of approximately 0.6496 among the three fragments studied (Fig. 3). In the dendrogram it can be seen that there are two groupings where the subfamily composition is more similar between the PNM and ALB fragments with 0.63 (Fig. 3).

The results obtained by means of the Kruskal–Wallis test determined that richness (H = 8.22, gl = 2, p = 0.015) and abundance (H = 12.95, gl = 2, p < 0.05) of braconid subfamilies observed were statistically different between the fragments. The results obtained by means of the Kruskal–Wallis test determined that richness (H = 6.053, gl = 2, p < 0.05) and abundance (H = 12.95, gl = 2, p < 0.05) of braconid morphospecies observed were statistically different between the fragments.

**Fig. 4:**
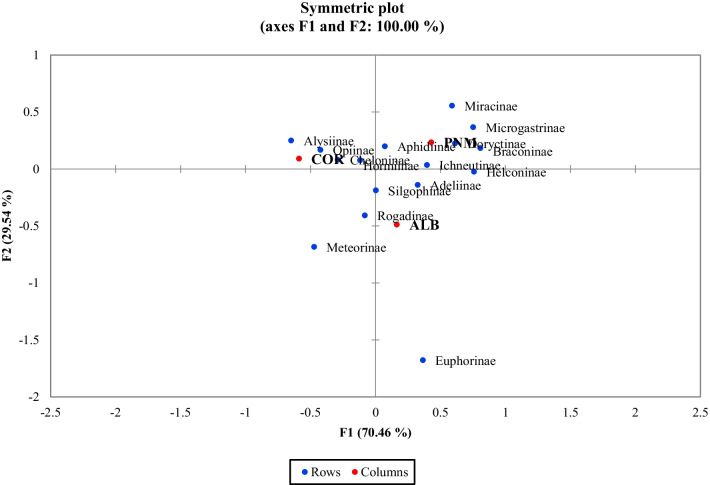
Correspondence analysis (CA) to compare the similarity of braconid wasp communities between habitat remnants of Corozal (COR), Albrook (ALB) and Metropolitan Natural Park (PNM). The two replicates of each habitat remnant are enveloped to make similarities among habitat remnants more apparent

In the correspondence analysis (CA), 100% cumulative percentage was explained for both morphospecies and subfamilies in the first two axes. A total inertia of 0.286 was calculated for braconid subfamilies and a total inertia of 0.469 for braconid morphospecies. Wasp assemblages (both morphospecies and subfamilies) were more similar between the ALB and PNM fragment. The data obtained demonstrated distinct clustering in the three fragments, as depicted in Fig. 4 for subfamilies. The data also demonstrated that the subfamily Euphorinae was only present in the fragment of ALB and Meteorinae was present in the remnants of ALB and COR.

**Fig. 5:**
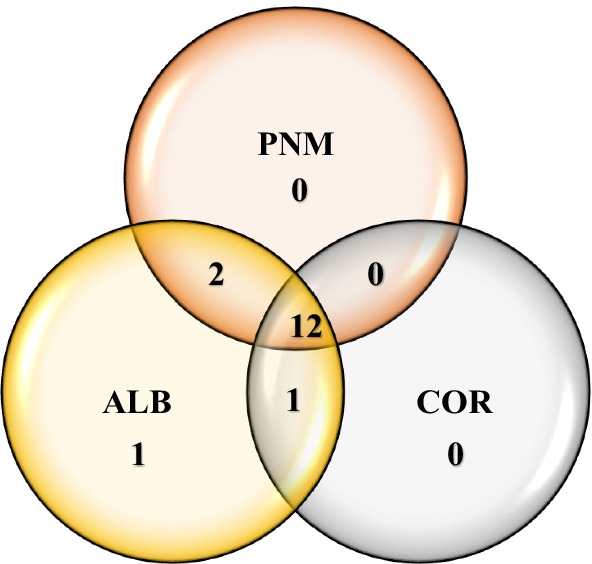
Venn diagram of the number of shared subfamilies of braconid wasps in the three fragments studied in lowland tropical forests of Panama: Corozal (COR), Albrook (ALB) and Metropolitan Natural Park (PNM). Of the 16 subfamilies, 12 were shared between the three habitat fragments studied

**Fig. 6:**
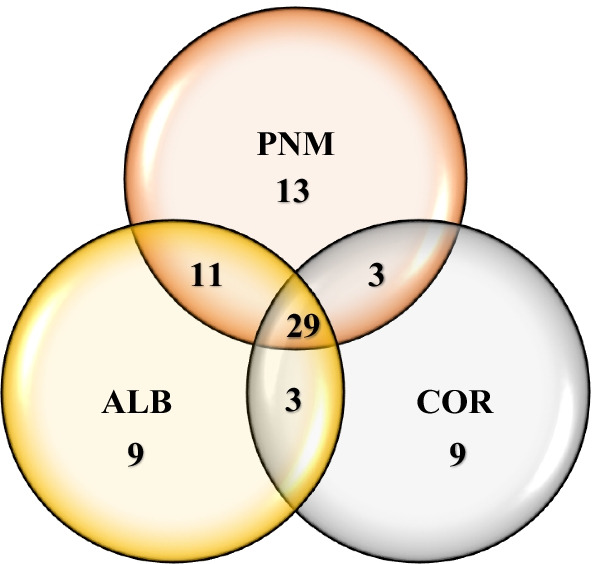
Venn diagram of the number of shared morphospecies of braconid wasps in the three fragments studied in lowland tropical forests of Panama: Corozal (COR), Albrook (ALB) and Metropolitan Natural Park (PNM). Of the 77 morphospecies, 29 were shared between the three habitat fragments studied

Of the 16 subfamilies, 12 were shared between all fragments studied, two between the ALB and PNM fragments, and one between the ALB and COR fragments (Fig. 5). One subfamily was registered in the fragment of ALB which was not shared between the fragments studied. Of the 77 morphospecies, 29 were shared between all fragments, 11 between the ALB and PNM fragments, three between the COR and PNM fragments and three between the ALB and COR fragments (Fig. 6). Nine registered morphospecies were only found in the ALB fragment, nine different morphospecies were only found in the COR fragment and 13 different morphospecies were found in the PNM fragment, all of which were not shared between the fragments.

According to the Diserud-Odegaard Index, a similarity of 0.9418 (94%) was estimated for subfamily composition in the fragments studied. Likewise, a similarity of 0.7401 (74%) was estimated for morphospecies composition in the fragments studied. These results depicted similar subfamily and morphospecies composition within and between the fragments studied.

## Discussion

Braconid wasp communities in fragmented lowland tropical forests of Panama are still very similar, both at the subfamily and morphospecies level. The fragments studied are considered to be areas large enough to maintain the biodiversity of wasp communities. The results of this study are consistent with those obtained by Valdés [31] and Ruiz-Guerra et al. [29]. Ruiz-Guerra et al. [29] demonstrated that Braconidae communities are very similar in the remnants of conserved tropical forests and secondary forests of Mexico. Valdés [31] depicted that butterfly communities are very similar in lowland tropical forests of Panama. In the study by Valdés [31], three of the four habitat fragments compared correspond to those used in the present study. The results of Valdés [31], indicated that when these three sites were compared, a similarity of 97% was obtained for the butterfly community. The proposed explanation stems from the species’ dispersal ability; since these taxa have the ability to fly, these organisms are able to move freely between fragments and as a result maintain high similarities between communities.

Richness and abundance of braconid subfamilies were statistically different between the fragments studied. The higher richness of braconid wasps found in the fragment of ALB can be explained by the heterogeneous vegetation, composed of open grassland, stubble, border vegetation and secondary forests in this fragment. The higher abundance and heterogeneity in the fragment of PNM can be explained by its proximity to a source; such as the remainder of the PNM. This is seen with the island-continent model, where local populations may be unequal in size and longevity, with one large fragment from which dispersers migrate to other fragments [32, 33]. The PNM habitat fragment is a part of the PNM; however, it has been isolated due to the development of highways. The habitat fragment however, is still considered a part of the PNM; where the remainder of the park is considered as the continent/source and the habitat fragment as the island/sink. Both fragments offer a wide range of microhabitats for organisms, allowing the survival of more individuals and increasing the availability and diversity of hosts [22]..The low richness in the fragment of COR demonstrates the introduction of biodiversity loss due to the process of fragmentation. This highlights the importance of maintaining continuous forests close to other remnants and the need to conserve fragments which provide various habitats for maintenance of species diversity.

The theory provided by the finding of braconid wasp communities is that of metapopulations [34], which assumes that species are distributed over a heterogenous space and not all territories are habitable for each species; as seen with the subfamilies of Euphorinae, Braconinae, Helconinae and Meterorinae. The fragments studied were divided into remnants/patches at a given moment primarily due to the effects of urbanization and industrialization, which separated the populations of braconid wasps forming their metapopulations. The model of the theory that supports this finding is that of patched populations [32, 34], where there are similar patches without clear distinction between sources and sinks. Two suggestions for which there is no distinction may be that the fragments have the same probability of being colonized by braconid wasps due to their dispersal ability and all fragments studied were of similar size. The fragments studied are considered to be areas large enough to keep the metapopulations of wasps in equilibrium. However, it would be necessary to prove how the dispersal dynamics of braconid wasps is carried out among fragments to prove that the fragments are indeed patched populations and determine the minimum critical size of the ecosystem to preserve the diversity and composition of species.

The results of this study also indicate that the communities of wasps (Hymenoptera: Braconidae) in the lowland forest fragments of Panama are mainly constituted by the subfamilies of Rogadinae, Alysiinae, Adeliinae, Doryctinae, Aphidiinae and Microgastrinae. The presence of these subfamilies is noteworthy since it implies interspecific relationships by parasitism between the species and other arthropods and implies that the fragments are diverse. The Microgastrinae, Adeliinae and Rogadinae subfamilies indicate interspecific relationships with Lepidoptera as they are endo parasitoids of larvae of the order Lepidoptera [35]. The Alysiinae subfamily indicates interspecific relationships with Diptera as they are endo parasitoids of the larvae or eggs of the order Diptera [35]. The Aphidiinae subfamily indicates interspecific relationships with the Stenorrhyncha order, particularly the Aphididae family [35]. The Doryctinae subfamily indicates both interspecific relationships and diversification in the fragments since they are ecto parasitoids of the larvae of the orders Coleoptera, Lepidoptera, Hymenoptera, Embioptera and Phytophagia [35]. The Doryctinae subfamily has been reported to dominate in a dry tropical forest in Mexico [36] and in a semi-deciduous forest in Venezuela [37].

There are two major limitations in this study that could be addressed in future research. First, although the appropriate trap was used, it is recommended to complement the research with another technique. An interesting technique for this community may be light traps [38, 39]. Species that are nocturnal are always attracted to light. Secondly, although the research demonstrated the composition of braconid wasp communities in three forest fragments, the research did not demonstrate the effect of habitat fragmentation on species communities. This can be demonstrated by comparing fragmented landscapes to a continuous forest or a gradient of fragment size.

**Fig. 7:**
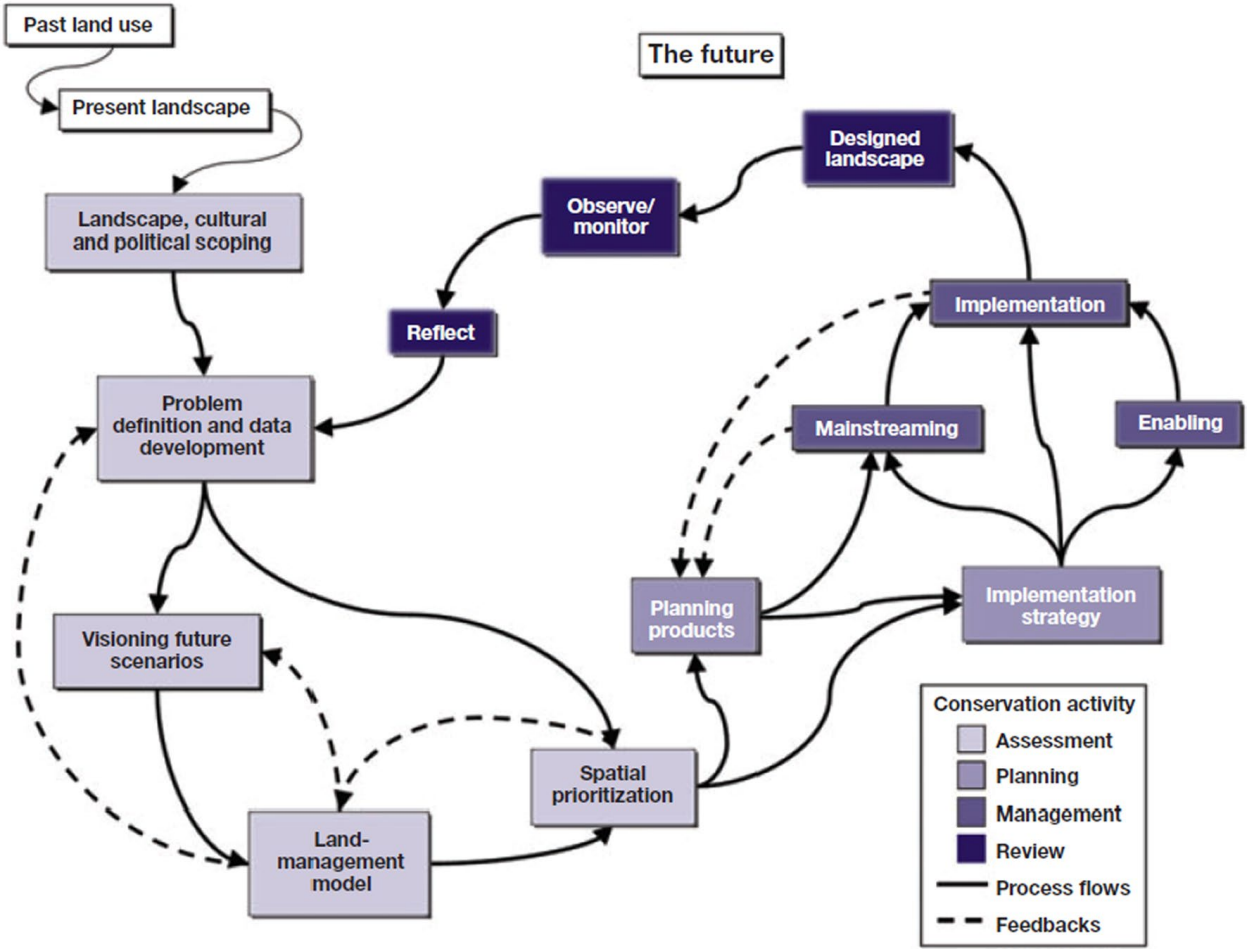
Operational model for conservation planning, incorporating assessment, and management phases (Grantham et al. 2010; Knight et al. 2006)

Taking this into account, effective planning for the conservation and preservation of the fragments studied require learning and adaptation. A substantial set of theoretical and practical guides have been developed to maintain biodiversity and ecosystem function [40], as well as operational models that use these guides for systematic conservation ([33]; Fig. 7). With the aid of the operational model for conservation planning by Knight et al. [41], it is suggested that the fragments be considered as stepping stones. Stepping stones can improve the persistence of metapopulations allowing the flow of individuals between fragments, ensuring the exchange of stochastic local extinction and recolonization [42]. The preservation of these fairly conserved fragments, with heterogenous vegetation, can favor the presence of organisms by offering various microhabitats to ensure the viability of metapopulations. Baum et al. [43] showed that a matrix can determine if, and to what extent, corridors and stepping stones, increase the connectivity of a landscape for the survival of species using *Prokelisia crocea* and *Spartina pectinata* as indicators. Spatial configuration is particularly important for regional dynamics and must be taken into account in management plans. Small and medium fragments play a fundamental role; in such a way that it is necessary to identify and preserve these fragments.

## Conclusions

The results of this study indicate that the richness, abundance, and composition of braconid wasps differ among habitat fragments in lowland tropical forests of Panama. Forest fragments have relatively high similarity of braconid wasp subfamilies and morphospecies. This finding was interpreted as an indication that a species’ dispersal ability plays a major role in its survival.

Annual inventories to assess the real change in braconid wasp communities or other organisms could provide critical monitoring of the effects of habitat fragmentation. These results can help to increase the understanding of the influence of habitat fragmentation on braconid wasp communities in Panama to develop successful conservation strategies. This would make it possible to address the question of the effect of fragmentation on the braconid wasp species.

## Methods

### Study sites

**Fig. 8:**
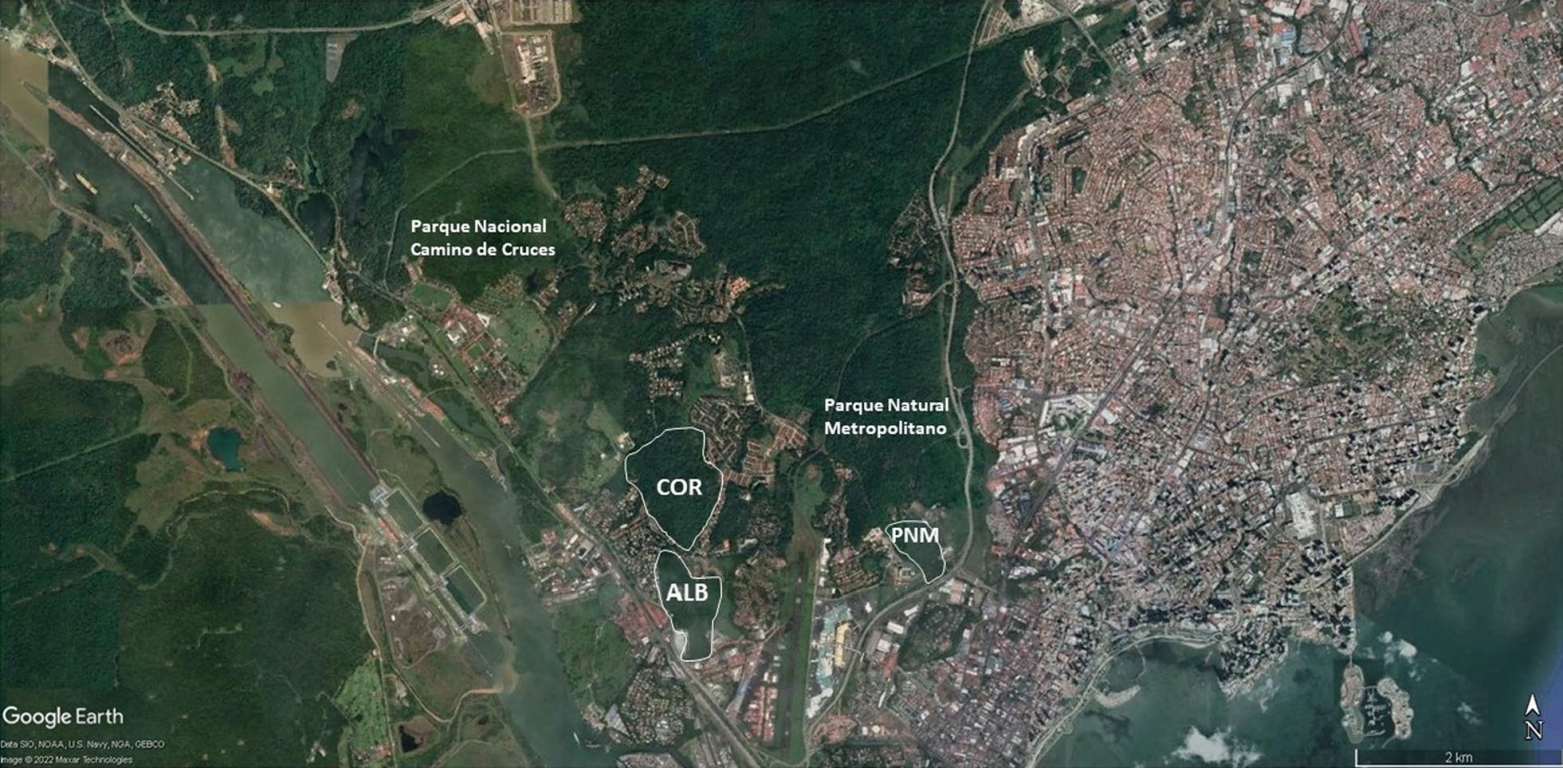
Map of the Pacific Basin of the Panama Canal where the habitat fragments studied in lowland tropical forests of Panama are located: Corozal (COR), Albrook (ALB) and Metropolitan Natural Park (PNM). Google Earth was used in order to construct the map

The three selected study sites in Panama City were Metropolitan Natural Park (PNM), public land in the town of Albrook (ALB) and public land in the town of Corozal (COR) (Fig. 8). Each fragment was bordered and labeled; as depicted in the map constructed with Google Earth. The PNM fragment is situated 8°59′41.55′′N and 79°32′35.22′′ W with an area of approximately 18.12 ha and a perimeter of 1.756 km. Vegetation is characterized as a mixture of tropical humid forest and lowland tropical dry forest, with few areas of stubble and grasslands, and a well-defined stratum. The ALB fragment is situated 8°58′37.49′′N and 79°33′43.82′′W with an area of approximately 34.79 ha and a perimeter of 5.003 km. Vegetation is characterized as heterogenous, composed of open grasslands, stubble and secondary forests. The COR fragment is situated 8°59′19.34′′N and 79°34′11.83′′W with an area of approximately 56.31 ha and a perimeter of 3.028 km. Vegetation is characterized as herbaceous with late secondary forest and some open areas. In this study, the habitat fragmentation definition defined by Saunders et al. [44] was used, where they described a fragment as any patch of native vegetation around which most of the original vegetation has been removed. For this reason, well-preserved fragments that are separated by a matrix of urban areas were considered as fragments in Panama City. The selected fragments are close in proximity to each other, which guarantees that the results obtained are effects of the fragmentation process of an original matrix and not the natural result caused by the distances between them. The distance between the fragments of ALB and COR is 1.129 km. The distance between the fragments of COR and PNM is 2.565 km. The distance between the fragments of ALB and PNM is 2.329 km.

The study sites form part of a biological corridor that runs along the eastern bank of the Panama Canal [45]. In accordance with the UNESCO classification, the three study sites are characterized as lowland tropical semi-deciduous forests. The three sites present annual average temperatures of 26.4 °C, with an annual average precipitation between 1501 to 1800 mm and altitudes between 20 to 150 masl. In the last 171 years, the forests along the eastern bank of the Panama Canal have been pressured by anthropic activities [46]. One of the first anthropic impact along the banks of the Panama Canal was the construction of Panama’s railway in 1850. The second anthropic impact along the banks of the Panama Canal was the construction of the French Canal in 1881. Nevertheless, the greatest anthropic impact started in the 1900’s when the forests along the eastern bank of the Panama Canal were intervened by military bases [46]. During this time, the remaining fragments maintained advanced secondary forest vegetation. In the last 50 years, these sites have been anthropically pressured due to the construction of neighborhoods along the banks of the Panama Canal [46]. The COR fragment has been used for Panama government security training activities. The ALB fragment has minimal human traffic; however, it contains a water reservoir at the peak of the fragment. The PNM fragment is along a hiking trail in a protected area but with visitor traffic [47].

### Sampling protocol

Two permanent lightweight Malaise Trap, Townes Style separated by approximately 0.2 km were installed in the center of each fragment and wasp samples were collected weekly from May 2019 to March 2020. The traps were made with organza fabric, with dimensions of 5.8 ft tall by 5.4 ft long, and contained a polyethylene collector bottle with 95% alcohol at its highest end [48]. This is a trap designed to collect fast-flying insects whose behavior is to fly upwards when it touches a surface. The Malaise Trap, Townes Style has been widely used in braconid wasps diversity studies in Central America and the world [49–53]. A reasonable flat, log-less area of approximately 2 m^2^ was chosen for the placement location of each trap in the forest. The collection bottle was placed facing the magnetic north and half-filled with 95% ethanol. The traps were placed in open areas which served as insect corridors. To exclude the edge effect, a safety distance of approximately 0.2 km was defined from the border of each fragment towards the center. Samples were collected every 7 days; resulting in a sampling effort of 44 weeks per trap for the period between 2019 and 2020.

Samples were taken to the Master’s degree laboratory at the University of Panama, where braconid specimens were separated from the rest of the sample and placed in vials of one dran with 95% ethanol. All braconid individuals were mounted on entomological pins, number 2 or 3. Braconid individuals were first sorted as morphospecies (M) using the method of Oliver and Beattie [54]. Oliver and Beattie [54] demonstrated that comparisons can be made using morphospecies assemblages as long as each contain a unique identification. Samples were then identified to subfamily level using the Sharkey and Campos taxonomic key from the book Aguilar et al. [35] and the manual by Sharkey et al. [26].

### Statistical analyses

In order to measure the diversity of subfamilies and morphospecies over a special scale, alpha (α) and beta (β) diversity were calculated. α diversity is the species richness of a particular community that is consider to be homogeneous. Currently there are many indices to measure α diversity. In this study, to determine the α of each fragment, the Simpson dominance index (D′), Shannon-Weiner Index (H′), Equity index or Pielou equity (J′) and Margalef diversity index were used. D′ takes into account the most important species without considering the rest of the species.

$${D}^{^{\prime}}=1-\sum ({{p}_{i})}^{2},$$

where p_i_ is the proportional abundance of species _i_ (the number of individuals of species _i_ divided by the total number of individuals in the sample).

H' combines information on species richness and equity in what is called diversity or heterogeneity [55, 56]. The average degree of uncertainty is measured in predicting the species to which a given randomly chosen individual within a biotic community belongs.

$$H^{\prime}= -\sum_{i=1}{p}_{i} ln {p}_{i},$$

where ln is the natural logarithm and p_i_ is the proportional abundance of species _i_ (the number of individuals of species _i_ divided by the total number of individuals in the sample).

J′ is the maximum possible diversity for a given number of species that occurs if all species are present in equal numbers. Its value ranges from 0 to 1, so that 1 corresponds to situations where all species are equally abundant.

$${J}^{^{\prime}}=\frac{H{^{\prime}}}{{H}^{^{\prime}}max},$$

where H' max is the lnS, H’ is the Shannon-Weiner index.

Margalef diversity index estimates the biodiversity of a community based on the numerical distribution of the individuals of the different species based on the number of individuals in the analyzed sample.

$${D}_{Mg}=\frac{S-1}{\mathrm{ln}N},$$

where S is the number of species and N is the total number of individuals.

The β diversity is the diversity of species between communities. It is the degree of species replacement or biotic change through environmental gradients [57, 58]. The indices used to determine β diversity are approaches based on pairwise similarities. To determine the β of each fragment, the Whittaker and Bray–Curtis Indices were used. The Bray Curtis Index is a modified version of the Sorensen Index [59]

$${S}_{S}=\frac{2a}{2a+b+c},$$

where a is the number of species present in both samples, b is the number of species found in community A and c is the number of species found in community B. Whittaker index estimates the degree of species replacement or biotic change through environmental gradients [60]. The α and β diversity indices were estimated using the EstimateS and the “vegan” package from RStudio.

For analysis of the data, all samples were pooled monthly, resulting in one sample per site. A Kruskal–Wallis test was performed using the program STATISTICA [61], to determine differences between the number of individuals registered in each study site. Correspondence analysis (CA) calculated by the program XL-Stat [62] was used to characterize the braconid community based on the number of individuals and site. A comparison of the composition of the braconid community was calculated using formula for the Multiple Similarity Index by Diserud and Odegaard [63], as given by the Eq. (2):

$${\text{C}}_{\text{S}}^{\text{T}}\text{=}\frac{\text{T}}{\text{T-1}}\left(\text{1} - \frac{{\text{S}}_{\text{T}}}{\sum_{\text{i}}{{\text{a}}}_{\text{i}}}\right),$$

where a_i_ is the number of species in site A_i_, T is the number of sites and S_T_ = $\sum_{i}{a}_{i}-\sum_{i<j}{a}_{ij}+ \sum_{i<j<k}{a}_{ijk}\dots ,$ a_ij_ is the number of species shared by sites A_i_ and A_j_; and a_ijk_ is the number of species shared by sites A_i_, A_j_ and A_k_, etc. The multiple similarity index takes into consideration the information of species shared by two or more sites and avoids the problem of covariance between pairwise similarities [63], reducing the probability of Type II errors. The multiple similarity index avoids the loss of information concerning the number of species shared among three or more sites and the lack of independence between pairwise similarities due to the repetition of each site in several pairs as seen with β diversity indices [63]. The calculation of this index was done manually.

## Data Availability

The datasets used and/or analyzed during the current study are available from the corresponding author on reasonable request.

## Abbreviations

ALB: Albrook
CA: Corresponding analysis
COR: Corozal
FRAG: Fragmentation Index
M: Morphospecies
M_#: Morphospecies number
PNM: Metropolitan Natural Park
